# ADAM17 as a proteolytic rheostat linking inflammation, tissue repair, and failure of inflammatory resolution in liver injury and fibrosis

**DOI:** 10.3389/fphar.2026.1955615

**Published:** 2026-09-21

**Authors:** Xiaodan Jiang, Jinxin Ma, Zhejun Liu, Jiang Chen

**Affiliations:** 1 Suzhou TCM Hospital Affiliated to Nanjing University of Chinese Medicine, Suzhou, China; 2 Qingdao Municipal Hospital, Qingdao, China

**Keywords:** ADAM17, inflammatory resolution, liver fibrosis, liver injury, MerTK, proteolytic rheostat, TREM2

## Abstract

A disintegrin and metalloproteinase 17 (ADAM17) is a membrane-bound sheddase that regulates inflammatory signaling, tissue repair, and fibrotic remodeling by converting cell-surface proteins into soluble forms. Rather than viewing ADAM17 simply as a proinflammatory enzyme, this review frames it as a proteolytic rheostat that dynamically adjusts the balance between membrane-bound and soluble molecular states in the injured liver. Within this framework, apparently opposing effects of ADAM17 can be understood as substrate-, cell-, and disease-stage-dependent consequences of ectodomain shedding. We particularly highlight two independently observed patterns of macrophage receptor-state remodeling: insufficient MerTK shedding may preserve profibrotic MerTK/ERK/TGF-β1 signaling, whereas excessive TREM2 shedding may reduce efferocytic capacity and contribute to failed inflammatory resolution. We further discuss how ADAM17-mediated shedding reshapes hepatocyte–macrophage–HSC communication and how soluble shedding products may provide circulating readouts of these processes. This framework suggests that therapeutic strategies should move beyond broad ADAM17 inhibition toward context-, cell-, or substrate-selective modulation that limits pathogenic signaling while preserving inflammation-resolving and tissue-repair functions.

## 1 Introduction

Liver fibrosis is a relatively conserved wound-healing response to persistent liver injury, characterized by excessive extracellular matrix (ECM) deposition and progressive scar formation (Henderson et al., 2020; Kisseleva and Brenner, 2021). Although liver injury can arise from diverse etiologies, including metabolic dysfunction, alcohol use, cholestasis, chronic viral infection, drugs, and toxins, these conditions often converge on shared pathological processes during disease progression (Tacke and Zimmermann, 2014; Seki and Schwabe, 2015; Krenkel and Tacke, 2017).

Persistent hepatocyte injury and death lead to the release of damage-associated molecular patterns (DAMPs) and proinflammatory mediators, as well as increased oxidative stress. Together, these signals promote the recruitment of inflammatory cells and the activation of Kupffer cells and monocyte-derived macrophages (Garcia-Martinez et al., 2016; Hirsova et al., 2016; Garcia-Martinez et al., 2023). Although the early inflammatory response can support host defense and tissue repair (Duffield et al., 2005; Feng et al., 2023), persistent injury or failure to resolve inflammation can shift this response toward chronic inflammation. Macrophage-derived cytokines, chemokines, and profibrotic mediators then promote hepatic stellate cell (HSC) activation through macrophage–HSC crosstalk (Karlmark et al., 2009; Baeck et al., 2014). Activated HSCs subsequently differentiate into myofibroblasts, express increased levels of α-smooth muscle actin (α-SMA), and deposit excessive collagen and other extracellular matrix components, thereby contributing to sinusoidal remodeling and fibrosis progression (Olsen et al., 2011).

Recent advances in macrophage research have greatly improved our understanding of the immune microenvironment during liver injury. Although the traditional M1/M2 framework provided a useful starting point, single-cell studies have revealed substantially greater heterogeneity among Kupffer cells, monocyte-derived macrophages, and scar-associated macrophage populations characterized by Triggering receptor expressed on myeloid cells 2 (TREM2) and SPP1 expression (Krenkel and Tacke, 2017; Ramachandran et al., 2019; Fabre et al., 2023). Beyond cellular composition and activation states, macrophage function is also dynamically regulated by the proteolytic ectodomain shedding of cell-surface receptors.

ADAM17 (a disintegrin and metalloproteinase 17), initially identified as the TNF-α-converting enzyme, is a major ectodomain sheddase that cleaves a broad range of membrane-bound cytokines, receptors, and growth factor precursors (Black et al., 1997; Lambrecht et al., 2018). Its substrates include TNF-α, TNFR1/2, IL-6R, EGFR ligands, and the macrophage receptors MerTK and TREM2 (Thorp et al., 2011; Cai et al., 2020; Schumacher and Rose-John, 2022; Wang et al., 2023). By altering the balance between membrane-bound and soluble molecular forms, ADAM17 can regulate inflammatory signaling, tissue repair, macrophage efferocytosis, and communication among multiple cell types during the progression from liver injury to fibrosis (Moss et al., 1997).

This review examines how ADAM17-mediated ectodomain shedding influences the progression from liver injury to fibrosis, with particular emphasis on (1) the context-dependent shedding of TNF-α/TNFRs, IL-6R, EGFR ligands, and other substrates involved in inflammation and regeneration; (2) the effects of MerTK and TREM2 cleavage on macrophage efferocytosis, the resolution of inflammation, and fibrosis progression; (3) the role of the ADAM17 sheddome in coordinating communication among hepatocytes, macrophages, and HSCs; and (4) potential value of soluble products generated by ADAM17-mediated cleavage as biomarkers of liver fibrosis.

## 2 ADAM17 biology and context-dependent regulation in liver injury

### 2.1 Structure and maturation of ADAM17

ADAM17 is a type I transmembrane metalloprotease composed of an N-terminal prodomain, a catalytic metalloprotease domain, a disintegrin domain, a membrane-proximal domain, a transmembrane segment, and a cytoplasmic tail (Dusterhoft et al., 2019). It is synthesized in the endoplasmic reticulum as an inactive precursor, with the prodomain suppressing its catalytic activity (Zunke and Rose-John, 2017). During transit through the Golgi apparatus, furin-like proprotein convertases remove the prodomain, thereby generating mature ADAM17 (Figure 1). However, prodomain removal alone does not ensure efficient substrate cleavage. ADAM17-mediated shedding also depends on proper intracellular trafficking, stabilization at the cell surface, substrate accessibility, and release from endogenous inhibition.

**FIGURE 1:**
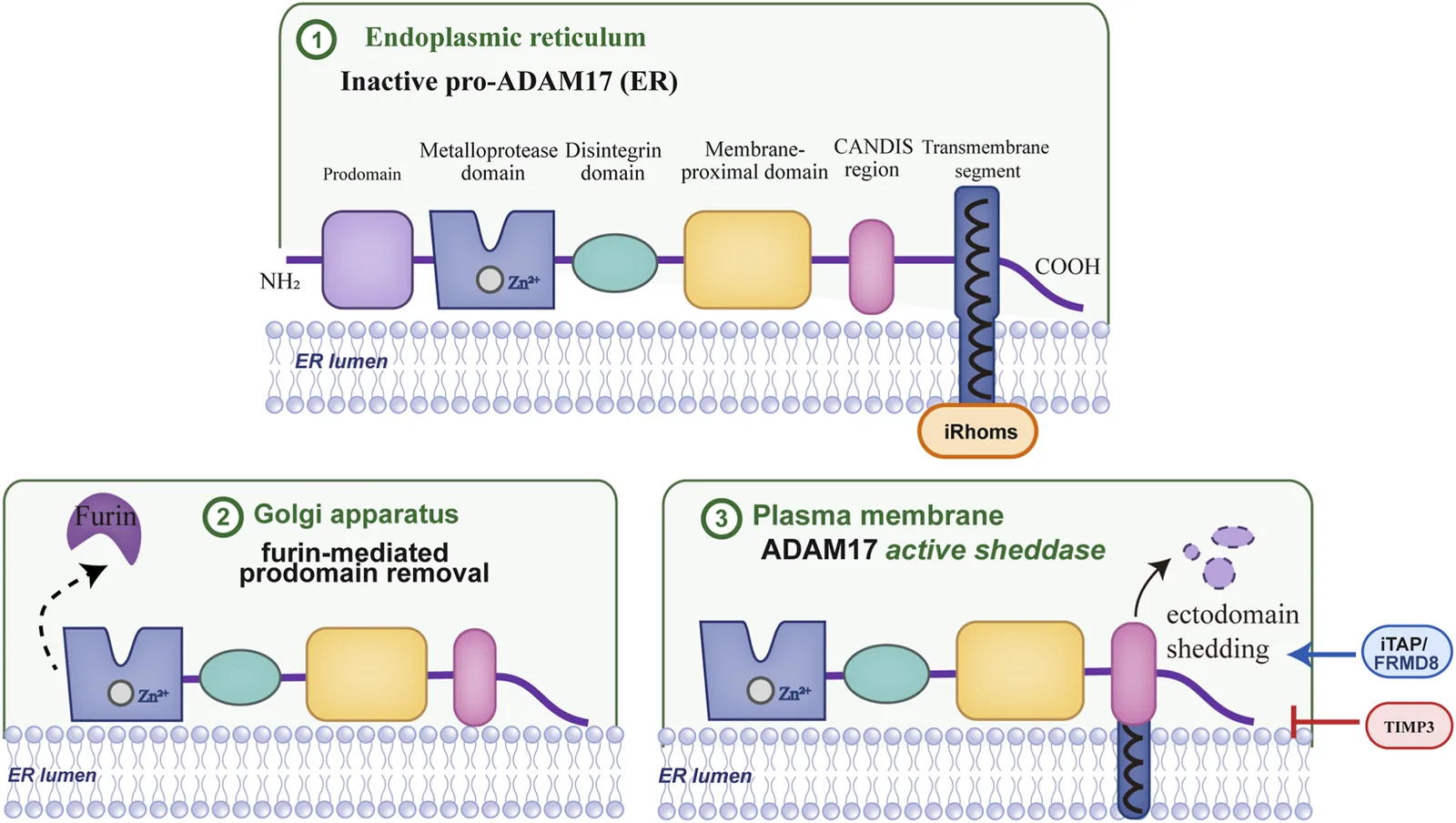
Maturation, trafficking, and cell-surface regulation of ADAM17. ADAM17 is synthesized as an inactive precursor in the endoplasmic reticulum (ER), trafficked with iRhom1/2 to the Golgi apparatus for prodomain removal, and subsequently delivered to the plasma membrane as a mature sheddase. Cell-surface ADAM17 is stabilized by the iRhom–iTAP/FRMD8 complex and negatively regulated by TIMP3.

### 2.2 Regulation of ADAM17 activity in the injured liver microenvironment

Beyond proteolytic maturation, ADAM17 sheddase activity is regulated by intracellular trafficking, the stability of the sheddase complex, localization at the plasma membrane, substrate accessibility, and endogenous inhibitors. Therefore, ADAM17 transcript levels or total protein abundance do not necessarily reflect its functional activity, which is more closely determined by the amount and activation state of mature ADAM17 at the cell surface. The pseudoproteases iRhom1 and iRhom2 are key regulators of this process. By forming sheddase complexes with ADAM17, they promote its exit from the endoplasmic reticulum, proteolytic maturation in the Golgi apparatus, and subsequent delivery to the plasma membrane (Cavadas et al., 2017). iRhoms also help determine the substrate selectivity of stimulus-induced ADAM17-mediated shedding (Maretzky et al., 2013; Li X. et al., 2015). The ADAM17–iRhom complex is further stabilized by iTAP/FRMD8, which prevents its lysosomal degradation and thereby preserves mature ADAM17 at the cell surface (Cavadas et al., 2017; Grieve et al., 2017; Kunzel et al., 2018; Oikonomidi et al., 2018).

TIMP3 is a major endogenous inhibitor of ADAM17 and restrains the release of cytokines, cytokine receptors, and growth factor precursors (Wisniewska et al., 2008). An imbalance between TIMP3 and ADAM17 could enhance ADAM17-dependent shedding during chronic liver injury, thereby promoting persistent inflammatory signaling, dysregulated regenerative responses, and maladaptive tissue remodeling (Lora et al., 2021).

In the injured liver, ADAM17 sheddase activity may be enhanced by inflammatory cytokines, Toll-like receptor signaling, oxidative stress, lipotoxicity, and other injury-associated stimuli. These signals can regulate ADAM17 through pathways involving protein kinase C, MAPK, reactive oxygen species, and changes in plasma membrane organization (Calligaris et al., 2021). Substrate cleavage is also influenced by local phosphatidylserine exposure (Sommer et al., 2016), redox conditions (Wang et al., 2009), and substrate accessibility (Le Gall et al., 2010) at the cell surface.

Importantly, changes in total ADAM17 activity do not necessarily produce equivalent shedding of all available substrates. Substrate selection is likely to depend on the local abundance and colocalization of ADAM17 and its substrates at the plasma membrane, stimulus-dependent regulation of the iRhom–ADAM17 complex, phosphatidylserine exposure and membrane organization, and the accessibility of individual juxtamembrane cleavage regions. iRhom2 phosphorylation is particularly relevant to stimulated ADAM17 shedding and may contribute to stimulus-dependent substrate selection. These mechanisms provide a potential explanation for why the same increase in ADAM17 activity can generate different shedding profiles across cell types and injury states. However, quantitative competition among ADAM17 substrates has not been systematically defined in hepatic cells.

### 2.3 ADAM17 as a proteolytic rheostat of membrane and soluble molecular states

At the plasma membrane, mature ADAM17 does not function as a simple on–off switch. Rather, it acts as a proteolytic rheostat by continuously shifting the balance between membrane-bound and soluble forms of its substrates. This process can alter not only whether a signaling pathway is active, but also its magnitude, duration, and spatial range (Sternlicht et al., 2005; Swendeman et al., 2008; Lin et al., 2022). Importantly, the biological output of ADAM17 cannot be inferred from its overall activity alone, because simultaneous shedding of different substrates may generate opposing consequences. For example, cleavage of tmTNF-α can broaden proinflammatory TNF signaling, whereas shedding of TNFR1/2 can generate soluble receptors that restrict the same pathway. Similarly, altered shedding of macrophage receptors can either deplete protective surface receptors or preserve maladaptive membrane signaling. We therefore use the term “proteolytic rheostat” to describe the substrate-, cell-, and stage-dependent redistribution of membrane and soluble molecular states, rather than merely the context-dependent effects of ADAM17. Within this framework, liver injury outcomes are determined by the pattern and balance of substrate shedding, not simply by an increase or decrease in total ADAM17 expression or activity.

An additional dimension of the proteolytic rheostat is disease stage. For clarity, we distinguish several overlapping phases of liver injury: acute injury, inflammatory resolution and regeneration, persistent inflammation with fibrogenic activation, established fibrosis, and fibrosis regression. These phases are not strictly sequential, but they provide a useful framework for interpreting apparently opposing effects of ADAM17. During acute injury, shedding may rapidly regulate inflammatory amplification and hepatocyte survival; during resolution and regeneration, receptor and growth-factor processing can support clearance of damaged cells and tissue repair. With persistent injury, the same pathways may acquire maladaptive functions that sustain inflammation or HSC activation. During fibrosis regression, restoration of macrophage clearance and remodeling programs may again become dominant. Thus, the biological consequence of ADAM17-mediated shedding should be interpreted according to both substrate identity and disease stage.

## 3 Shedding of inflammatory and regenerative substrates

### 3.1 TNF-α and TNFR1/2: simultaneous amplification and restriction of TNF signaling

TNF-α is synthesized as a transmembrane precursor (tmTNF-α) that is predominantly cleaved by ADAM17 to generate soluble TNF-α (sTNF-α). Whereas tmTNF-α mainly mediates contact-dependent and bidirectional signaling, sTNF-α acts over a broader spatial range and amplifies inflammatory responses. ADAM17 also sheds TNF receptor 1 (TNFR1) and TNF receptor 2 (TNFR2), generating sTNFRs that attenuate TNF signaling (Li et al., 2019).

ADAM17 can exert context-dependent effects on TNF signaling during liver injury because it cleaves both tmTNF-α and its receptors. Cleavage of tmTNF-α releases sTNF-α, which can amplify hepatic inflammation and tissue injury. In hepatic ischemia–reperfusion injury, ADAM17 expression and activity are increased in parallel with TNF-α, TNFR1, and IL-6 levels, whereas TIMP3-mediated inhibition of ADAM17 reduced circulating TNF-α and liver injury (Tang et al., 2006). In a chronic PM2.5 exposure model, increased hepatic iRhom2 and ADAM17 expression was associated with elevated TNF-α, TNFR2, and other inflammatory mediators, as well as hepatic steatosis and dyslipidemia (Ge et al., 2017). By contrast, ADAM17-mediated shedding of TNFR1 and TNFR2 generates soluble receptors that can bind TNF-α and limit excessive TNF signaling (Xanthoulea et al., 2004). In a sepsis model, the iNOS/NO/cGMP signaling axis promotes ADAM17-mediated shedding of TNFR1 from hepatocytes, thereby increasing circulating sTNFR1 levels and limiting systemic inflammation and organ injury (Chanthaphavong et al., 2012). In Fas-induced fulminant hepatitis, Timp3 deficiency enhances ADAM17-mediated TNFR1 shedding and the release of EGFR ligands, collectively attenuating TNF/TNFR1 signaling while promoting hepatocyte survival (Murthy et al., 2010). In a murine bile duct ligation model, iRhom2 deficiency reduced ADAM17-dependent shedding of TNFR1 and TNFR2, exacerbating liver fibrosis and hepatic stellate cell activation (Sundaram et al., 2019). Viewed through the rheostat framework, the TNF axis illustrates how simultaneous cleavage of a ligand and its receptors can generate opposing outputs. The balance between these shedding events is likely to vary across disease stages. During acute inflammatory injury, rapid release of sTNF-α may amplify hepatocellular damage, whereas concomitant TNFR shedding can provide a counter-regulatory mechanism that limits excessive TNF signaling. With persistent inflammation, prolonged alterations in TNF/TNFR processing may contribute to sustained inflammatory and fibrogenic signaling.

### 3.2 ADAM17-mediated IL-6R shedding and trans-signaling

Interleukin-6 receptor (IL-6R) is a well-characterized substrate of ADAM17-mediated ectodomain shedding (Schumacher et al., 2015). ADAM17 cleaves membrane-bound IL-6R to generate soluble IL-6R (sIL-6R), which binds IL-6 to form a signaling complex capable of activating gp130 on cells that do not express membrane-bound IL-6R (Riethmueller et al., 2017; Zunke and Rose-John, 2017). This process, known as IL-6 trans-signaling, broadens the cellular range of IL-6 responsiveness (Yousif et al., 2021).

Circulating levels of IL-6 and sIL-6R are elevated in patients with MASH (Shipovskaya et al., 2025) and HCV-associated chronic liver disease (Migita et al., 2006) and have been associated with biochemical markers of liver injury, fibrosis indices, and impaired hepatic function. In alcohol-associated hepatitis, IL-6 trans-signaling sustains STAT3 activation in hepatocytes and promotes the expression of neutrophil-recruiting mediators, thereby contributing to intrahepatic neutrophilic inflammation (Sarode et al., 2026).

The functional consequences of IL-6 classical and trans-signaling are strongly dependent on cellular context, injury model, and disease stage. Although IL-6 trans-signaling is frequently associated with inflammatory amplification, experimental activation of trans-signaling can also support hepatocyte survival and regeneration in selected acute liver injury models. Experimental studies have used hyper-IL-6, a designer fusion protein composed of IL-6 covalently linked to sIL-6R, to selectively activate trans-signaling (Fischer et al., 1997). In these models, hyper-IL-6 reduced hepatocellular injury and promoted regenerative responses after acetaminophen- or D-galactosamine-induced injury (Galun et al., 2000; Li S. Q. et al., 2015). IL-6 trans-signaling also contributes to tissue repair and restoration of hepatic glycogen stores after CCl_4_-induced injury (Gewiese-Rabsch et al., 2010). Following partial hepatectomy, signaling mediated by the IL-6/sIL-6R complex accelerates hepatocyte proliferation, in part through cooperation with HGF and activation of PI3K/AKT signaling (Nechemia-Arbely et al., 2011). Moreover, in a genetic model in which IL-6 trans-signaling was preserved despite the loss of classical signaling, mice exhibited near-normal survival and liver regeneration after partial hepatectomy. These findings suggest that trans-signaling can partially compensate for impaired classical IL-6 signaling during liver regeneration (Fazel Modares et al., 2019). Within the rheostat framework, these findings indicate that the consequences of IL-6R shedding are stage dependent. During acute injury and early regeneration, IL-6 trans-signaling can support hepatocyte survival and proliferative responses, whereas persistent trans-signaling in chronic inflammatory liver disease may contribute to sustained inflammatory activation.

### 3.3 EGFR ligands: balancing hepatocyte repair and fibrogenic activation

ADAM17 contributes to the shedding of several epidermal growth factor receptor (EGFR) ligands, including amphiregulin (AREG), heparin-binding EGF-like growth factor (HB-EGF), epiregulin, transforming growth factor-α (TGF-α), and epigen (Sahin et al., 2004; Sahin and Blobel, 2007). The released ligands can activate EGFR and downstream signaling pathways, thereby promoting cell proliferation and tissue repair (Liu et al., 2012).

AREG is synthesized as a membrane-bound EGFR ligand that can be released through ADAM17-mediated ectodomain shedding. AREG promotes graft regeneration after small-for-size liver transplantation, attenuates Concanavalin A-induced hepatotoxicity, and protects against cholestatic liver injury in BDL- and ANIT-treated mice (Liu et al., 2012; Santamaria et al., 2019; Wu et al., 2020). However, its effects appear to differ in chronic metabolic liver disease. In MASH, AREG-producing regulatory T cells accumulate in the liver, and Treg-specific Areg deficiency attenuates fibrosis by reducing EGFR-dependent profibrotic activation of HSCs (Savage et al., 2024). AREG has also been reported to increase the expression of inflammatory mediators through NF-κB and MAPK signaling in models of hepatic steatosis (Heo et al., 2023). Thus, ADAM17-mediated AREG release may support hepatocyte survival and regeneration during acute injury, whereas persistent AREG signaling may promote inflammation and HSC activation during chronic metabolic liver disease.

HB-EGF is synthesized as the membrane-anchored precursor proHB-EGF, which undergoes ADAM17-mediated ectodomain shedding to release mature soluble HB-EGF. Soluble HB-EGF activates EGFR and ErbB4 signaling and thereby regulates cell proliferation, survival, and tissue repair (Dao et al., 2018). In liver-specific proHB-EGF transgenic mice subjected to partial hepatectomy, hepatocyte proliferation, as assessed by BrdU labeling, was increased and recovery of liver mass was accelerated (Kiso et al., 2003). The early increase in soluble HB-EGF after hepatectomy was consistent with enhanced proHB-EGF shedding during the initial phase of liver regeneration. Following hepatic ischemia–reperfusion injury, eosinophil-derived IL-4 stimulated hepatic macrophages to produce HB-EGF, which in turn supported hepatocyte proliferation and liver regeneration (Yang et al., 2024). In CCl_4_-induced acute liver injury, liver-specific HB-EGF deficiency worsened hepatocellular injury and apoptosis (Takemura et al., 2013b), whereas hepatic HB-EGF gene transfer attenuated Fas-induced liver injury and enhanced hepatocyte regeneration (Khai et al., 2006). The effects of HB-EGF on fibrogenesis are also context dependent. HB-EGF can promote HSC proliferation through EGFR/ErbB4/ERK/Akt signaling *in vitro* (Zhang et al., 2014), whereas HB-EGF deficiency exacerbates fibrosis in BDL (Takemura et al., 2013a) and TAA-induced injury models, and exogenous HB-EGF can suppress activation-associated fibrogenic responses in primary HSC (Huang et al., 2012).

TGF-α is another EGFR ligand that may link ADAM17 activity to hepatocyte proliferation and liver regeneration. Following partial hepatectomy, the number of TGF-α-positive hepatocytes increased in parallel with hepatocyte proliferation (Okada et al., 2002). ADAM17 expression also increased progressively and was temporally associated with the increase in TGF-α-positive hepatocytes (Lin et al., 2008). These observations are consistent with, but do not establish, ADAM17-dependent TGF-α shedding during liver regeneration. In Fas-induced fulminant hepatitis, enhanced ADAM17-dependent shedding in the setting of TIMP3 deficiency increased the release of TGF-α, AREG, and HB-EGF and was associated with enhanced EGFR–ERK1/2 survival signaling. Through the shedding of these EGFR ligands, ADAM17 may coordinate death and survival pathways during acute hepatotoxic stress (Murthy et al., 2010), as summarized in Table 1 and illustrated in Figure 2.

**TABLE 1:** Major ADAM17 substrates implicated in liver injury and fibrosis and the strength of supporting evidence.

ADAM17 substrate	Consequence of shedding	Representative liver context	Major biological consequence	Strength of liver-specific evidence*	Key references
TNF-α	tmTNF-α → sTNF-α; increased signaling range	Hepatic I/R injury; chronic PM2.5 exposure; fulminant hepatitis	Broadens TNF signaling and may amplify acute inflammatory injury; biological output is modified by simultaneous TNFR shedding	Strong	Tang et al., 2006; Ge et al., 2017; Cao et al., 2022; Murthy et al., 2010
TNFR1/2	↓ Surface TNFR1/2; ↑ sTNFR1/2	Sepsis-associated liver injury; Fas-induced hepatitis; BDL-induced fibrosis	Soluble TNFRs can limit excessive TNF signaling; impaired receptor shedding may favor hepatotoxic and fibrogenic signaling	Strong	Xanthoulea et al., 2004; Chanthaphavong et al., 2012; Murthy et al., 2010; Sundaram et al., 2019
IL-6R	mIL-6R → sIL-6R; enables IL-6 trans-signaling	MASH and chronic HCV infection; alcohol-associated hepatitis; acute toxic injury; partial hepatectomy	Expands the cellular range of IL-6 signaling; may promote inflammation in chronic disease but support hepatocyte survival and regeneration in selected acute settings	Moderate	Schumacher et al., 2015; Galun et al., 2000; Gewiese-Rabsch et al., 2010; Nechemia-Arbely et al., 2011; Fazel Modares et al., 2019; Sarode et al., 2026
EGFR ligands (AREG, HB-EGF, TGF-α)	Release of soluble EGFR ligands	Partial hepatectomy; transplantation; ConA and Fas injury; cholestatic injury; MASH	Transient shedding can support hepatocyte survival and regeneration, whereas persistent or cell-specific EGFR signaling may promote inflammation and HSC activation	Moderate	Sahin et al., 2004; Kiso et al., 2003; Liu et al., 2012; Santamaria et al., 2019; Savage et al., 2024; Murthy et al., 2010
MerTK	↓ Surface MerTK; ↑ sMer; reduced shedding causes receptor retention	Acute hepatic I/R injury; fibrotic MASH	Excessive shedding can impair efferocytosis during acute injury, whereas insufficient shedding can sustain MerTK/ERK/TGF-β1 signaling and macrophage–HSC profibrotic communication	Strong	Thorp et al., 2011; Cai et al., 2020; Pastore et al., 2022; Guan et al., 2026
TREM2	↓ Surface TREM2; ↑ sTREM2	Prolonged hypernutrition-induced MASH; CCl_4_-induced fibrosis	Excessive shedding can reduce TREM2-dependent efferocytosis, promote accumulation of dying hepatocytes and DAMPs, and sustain inflammatory signaling	Strong in MASH; Moderate in other liver settings	Wang et al., 2023; Feuerbach et al., 2017; Ganguly et al., 2024; Shan et al., 2024
AXL	↓ Surface AXL; ↑ sAXL	MASH; advanced cirrhosis	May alter GAS6–TAM signaling and macrophage homeostasis; direct hepatic ADAM17-dependent causality remains insufficiently established	Limited	Tutusaus et al., 2020; Pop et al., 2023
CD40, ICAM-1, VEGFR2	Release of soluble ectodomains; ↓ surface receptor availability	Primarily non-hepatic endothelial systems; relevance to hepatic sinusoidal endothelial cells remains uncertain	Potential regulation of leukocyte adhesion, transendothelial migration, and vascular signaling; hepatic relevance is currently extrapolated	Limited	Tsakadze et al., 2006; Swendeman et al., 2008; Klersy et al., 2023

* Strength of liver-specific evidence was qualitatively assigned according to three considerations: liver specificity, mechanistic directness of the ADAM17–substrate relationship, and reproducibility across independent studies or disease models. “Strong” indicates direct liver-specific evidence linking ADAM17-mediated shedding to functional or pathological outcomes, supported by causal manipulation and/or multiple independent studies. “Moderate” indicates liver-specific evidence with mechanistic support but limited replication, incomplete causal attribution to ADAM17, or dependence on selected experimental models. “Limited” indicates predominantly associative, indirect, single-study, or non-hepatic evidence from which hepatic relevance is inferred. This grading is intended as a qualitative appraisal rather than a formal systematic evidence-rating framework.

**FIGURE 2:**
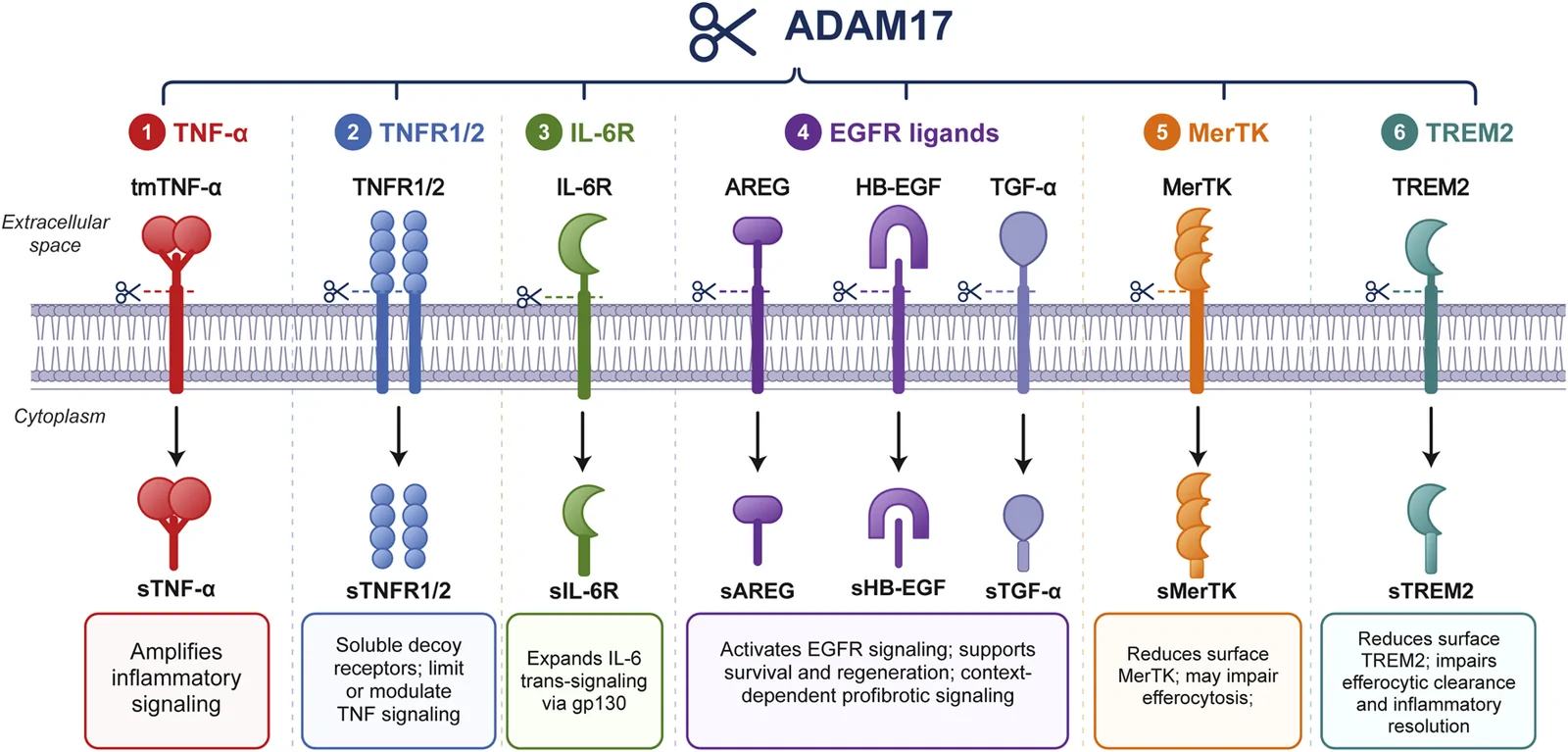
Functional consequences of ADAM17-mediated ectodomain shedding of major substrates involved in liver injury and fibrosis. ADAM17-mediated shedding converts membrane-bound TNF-α, TNFR1/2, IL-6R, EGFR ligands, MerTK, and TREM2 into soluble products, thereby altering inflammatory signaling, receptor availability, hepatocyte repair, efferocytosis, and fibrotic responses. The biological outcome depends on the substrate, cell type, and disease context.

The EGFR-ligand axis illustrates a clear stage-dependent shift in ADAM17 function. During acute injury and the regenerative phase, transient shedding of AREG, HB-EGF, and TGF-α can support hepatocyte survival and restoration of liver mass. In contrast, during persistent metabolic or fibrotic injury, sustained or cell-specific EGFR signaling may promote inflammatory activation and HSC proliferation. Thus, apparently opposing effects of EGFR-ligand shedding may reflect differences in timing, cellular target, and duration of signaling rather than contradictory functions of ADAM17 itself.

## 4 ADAM17-mediated remodeling of macrophage receptor states

### 4.1 Macrophage state transitions and inflammatory resolution

Inflammatory resolution in the injured liver is an active process that extends beyond the clearance of apoptotic cells. Efferocytosis is an important initiating component, because efficient removal of dying hepatocytes limits the secondary release of intracellular contents and damage-associated molecular patterns (DAMPs). However, successful resolution also requires a broader transition of hepatic macrophages from inflammatory or profibrotic states toward reparative programs that support debris clearance, lipid processing, matrix remodeling, and restoration of tissue homeostasis. During MASH regression, for example, TREM2-positive lipid-associated macrophages exhibit enhanced phagocytic, lipid-processing, and collagen-degrading programs and are associated with hepatic stellate cell (HSC) inactivation and fibrosis resolution. Thus, resolution involves not only termination of inflammatory signaling but also attenuation of macrophage–HSC profibrotic communication and reversal of the activated stromal state.

ADAM17-mediated receptor shedding may influence several components of this transition. By altering the surface availability of MerTK and TREM2, ADAM17 can modify macrophage efferocytic capacity and downstream signaling, potentially affecting whether macrophages remain in inflammation-sustaining states or acquire functions compatible with tissue repair. However, direct evidence that ADAM17 controls global macrophage state transitions, HSC inactivation, or fibrosis regression remains limited. These processes should therefore be viewed as an important extension of the ADAM17 receptor-state model that requires further mechanistic validation.

Efferocytosis is a specialized process through which macrophages and other phagocytes recognize, engulf, and degrade apoptotic cells (Boada-Romero et al., 2020). In the injured liver, efficient efferocytosis prevents the secondary release of intracellular contents and damage-associated molecular patterns, thereby promoting inflammatory resolution (Krenkel and Tacke, 2017). This process is mediated by several receptor systems, including TAM receptors, TREM2, TIM family members, scavenger receptors, and integrins (Ni et al., 2021; Hu et al., 2023). Among these receptors, MerTK and TREM2 are particularly relevant to ADAM17-dependent regulation in liver disease.

ADAM17-mediated ectodomain shedding reshapes the abundance and functional state of receptors at the macrophage surface. Cleavage of MerTK and TREM2 reduces the availability of functional membrane-bound receptors while generating soluble ectodomains (Thorp et al., 2011; Feuerbach et al., 2017). In liver disease, these changes may affect not only macrophage efferocytic capacity but also downstream inflammatory and profibrotic signaling (Cai et al., 2020; Wang et al., 2023). Importantly, the pathological consequences of shedding differ between the two receptors, and their shedding states may vary according to the stage and context of liver injury. The following sections therefore examine MerTK and TREM2 as two distinct patterns of ADAM17-dependent macrophage receptor-state imbalance.

### 4.2 The MerTK axis: from efferocytosis to a profibrotic switch

MerTK is a member of the TAM receptor tyrosine kinase family and an important efferocytic receptor expressed by hepatic macrophages (Triantafyllou et al., 2018; Chen et al., 2023). Through the bridging ligands Gas6 and Protein S, MerTK recognizes phosphatidylserine exposed on apoptotic cells and promotes their engulfment, thereby supporting inflammatory resolution, tissue homeostasis, and repair (Pastore et al., 2022). MerTK undergoes proteolytic ectodomain shedding by several proteases, among which ADAM17 is the best-characterized sheddase (Lahey et al., 2024). ADAM17-mediated cleavage reduces the abundance of cell-surface MerTK and releases sMer, altering both receptor availability and ligand-dependent signaling.

Inflammatory stimulation can enhance MerTK shedding. Exposure of macrophages to lipopolysaccharide or phorbol 12-myristate 13-acetate promotes MerTK ectodomain shedding and sMer release (Sather et al., 2007). Mechanistic studies have shown that lipopolysaccharide-induced MerTK shedding requires ADAM17 activity (Thorp et al., 2011). In addition to serving as a marker of cell-surface MerTK loss, sMer can bind and sequester Gas6, thereby functioning as a soluble decoy receptor that limits ligand-dependent activation of the remaining membrane-bound MerTK (Sather et al., 2007; Thorp et al., 2011). Thus, the simultaneous reduction in cell-surface MerTK and sequestration of its ligand may impair MerTK-dependent efferocytosis.

During acute liver injury, excessive MerTK shedding may compromise macrophage-mediated clearance of apoptotic cells. In a hepatic ischemia–reperfusion injury model, activation of the NLRP3–caspase-1 pathway increased ADAM17 activity and MerTK cleavage and was accompanied by reduced macrophage efferocytosis and aggravated inflammatory injury (Guan et al., 2026). These findings suggest that excessive ADAM17-mediated shedding reduces surface MerTK sufficiently to impair efficient apoptotic cell clearance. However, whether the same mechanism operates across other forms of acute liver injury remains to be established.

A contrasting pattern has been observed during the progression from hepatic steatosis to fibrotic MASH. In this setting, reduced ADAM17-mediated MerTK shedding in resident hepatic macrophages increases the retention of MerTK at the cell surface and sustains ligand-dependent signaling. Upon ligand binding, MerTK undergoes autophosphorylation, recruits growth factor receptor-bound protein 2, and activates ERK1/2 signaling (Cai et al., 2018). Sustained MerTK/ERK1/2 signaling increases macrophage production of TGF-β1, which acts on HSCs in a paracrine manner to activate SMAD2/3 signaling, induce COL1A1 expression, and promote extracellular matrix deposition (Cai et al., 2020). Consistent with this mechanism, conditioned medium from Gas6-stimulated MerTK-positive macrophages increased the expression of profibrogenic genes, including TIMP1, ACTA2, and COL1A1, in hepatic stellate cells (Pastore et al., 2022).

These findings indicate that the functional effects of MerTK are determined not simply by its total expression, but by the dynamic balance between cell-surface retention and ectodomain shedding. Excessive shedding during acute injury can deplete cell-surface MerTK and impair efferocytic clearance, whereas insufficient shedding during chronic MASH can sustain MerTK/ERK/TGF-β1 signaling and promote macrophage–HSC profibrotic communication.

### 4.3 The TREM2 axis: efferocytic failure under lipid stress

TREM2 is a type I transmembrane receptor expressed predominantly by myeloid cells (Walter, 2016). In the liver, membrane-bound TREM2 promotes macrophage clearance of lipid-laden apoptotic hepatocytes and helps contain inflammation during metabolic stress (Ganguly et al., 2024). During early steatotic injury, sphingosine-1-phosphate released by lipid-laden apoptotic hepatocytes induces TREM2 expression in infiltrating macrophages, enhancing efferocytosis and limiting secondary inflammatory activation (Wang et al., 2023). Consistent with this protective role, TREM2 deficiency impairs apoptotic cell clearance and exacerbates hepatic inflammation and fibrosis in fatty liver disease (Liebold et al., 2023). TREM2 also appears to contribute to tissue repair during disease regression. During MASH regression, TREM2-positive lipid-associated macrophages also exhibit enhanced phagocytic, lipid-processing, and collagen-degrading programs and are associated with hepatic stellate cell inactivation and fibrosis resolution (Ganguly et al., 2024). Collectively, these findings suggest that TREM2-dependent macrophage functions contribute to both the containment of inflammation during liver injury and tissue remodeling during recovery.

The protective activity of mTREM2 is regulated by ectodomain shedding. TREM2 is cleaved within its stalk region at the His157–Ser158 peptide bond, releasing sTREM2 and reducing the abundance of full-length receptor at the cell surface (Schlepckow et al., 2017; Thornton et al., 2017). Both ADAM10 and ADAM17 can mediate TREM2 shedding (Schlepckow et al., 2017). However, pharmacological and genetic studies have identified ADAM17 as the predominant stimulus-responsive TREM2 sheddase in human cellular systems (Feuerbach et al., 2017). Following ectodomain shedding, the remaining membrane-associated TREM2 C-terminal fragment is further processed by γ-secretase, thereby completing the proteolytic processing of the receptor and preventing signaling by full-length TREM2 (Wunderlich et al., 2013).

Direct evidence linking enhanced TREM2 shedding to defective macrophage clearance has been obtained in a prolonged hypernutrition model of MASH. Increased hepatic levels of TNF-α and IL-1β promoted ADAM17-dependent TREM2 cleavage, reducing surface TREM2 on macrophages and impairing the efferocytosis of lipid-laden apoptotic hepatocytes. Defective clearance led to the intrahepatic accumulation of dying hepatocytes, which further increased inflammatory cytokine production and reinforced TREM2 shedding. Evidence from other liver injury models further supports the protective importance of intact TREM2 signaling. In CCl_4_-induced fibrosis, TREM2 deficiency impairs macrophage phagocytic activity, increases the accumulation of dying hepatocytes and mitochondrial DAMPs, and aggravates inflammation and fibrosis (Shan et al., 2024).

Overall, excessive ADAM17-mediated TREM2 shedding reduces the surface availability of a protective macrophage receptor and promotes efferocytic failure under prolonged metabolic stress. This mechanism differs from the MerTK axis, in which insufficient shedding can preserve sustained profibrotic membrane signaling. Thus, dysregulated ADAM17 activity may generate two contrasting macrophage receptor states: loss of TREM2-dependent clearance capacity and persistence of MerTK-dependent profibrotic signaling.

### 4.4 MerTK and TREM2 shedding as distinct patterns of macrophage receptor-state remodeling

Available studies identify two distinct patterns of ADAM17-associated macrophage receptor remodeling, although these patterns have been demonstrated in different experimental contexts and should not be considered components of a single coordinated mechanism. During the progression from hepatic steatosis to fibrotic MASH, reduced MerTK shedding increases cell-surface receptor retention and can sustain MerTK/ERK/TGF-β1 signaling, thereby promoting macrophage–HSC profibrotic communication. In contrast, during prolonged hypernutrition, enhanced ADAM17-dependent TREM2 shedding reduces surface TREM2 and impairs the clearance of lipid-laden dying hepatocytes, contributing to persistent inflammatory signaling.

The value of comparing these pathways therefore lies in illustrating a common regulatory principle rather than proposing that they necessarily coexist. Pathological macrophage states may arise from opposite deviations in substrate-specific shedding: insufficient MerTK shedding can preserve maladaptive membrane signaling, whereas excessive TREM2 shedding can deplete a receptor required for efficient efferocytic clearance. The MerTK–TREM2 comparison thus provides an example of the proteolytic rheostat concept, in which the biological consequence of ADAM17 activity depends on the substrate-specific balance between membrane retention and ectodomain release rather than on overall ADAM17 activity alone (Figure 3). Whether these receptor-state imbalances occur simultaneously, sequentially, or in distinct macrophage populations, disease stages, or spatial niches remains unknown. Accordingly, this comparison should be regarded as a conceptual framework derived from separate lines of evidence rather than a demonstrated unified pathway.

**FIGURE 3:**
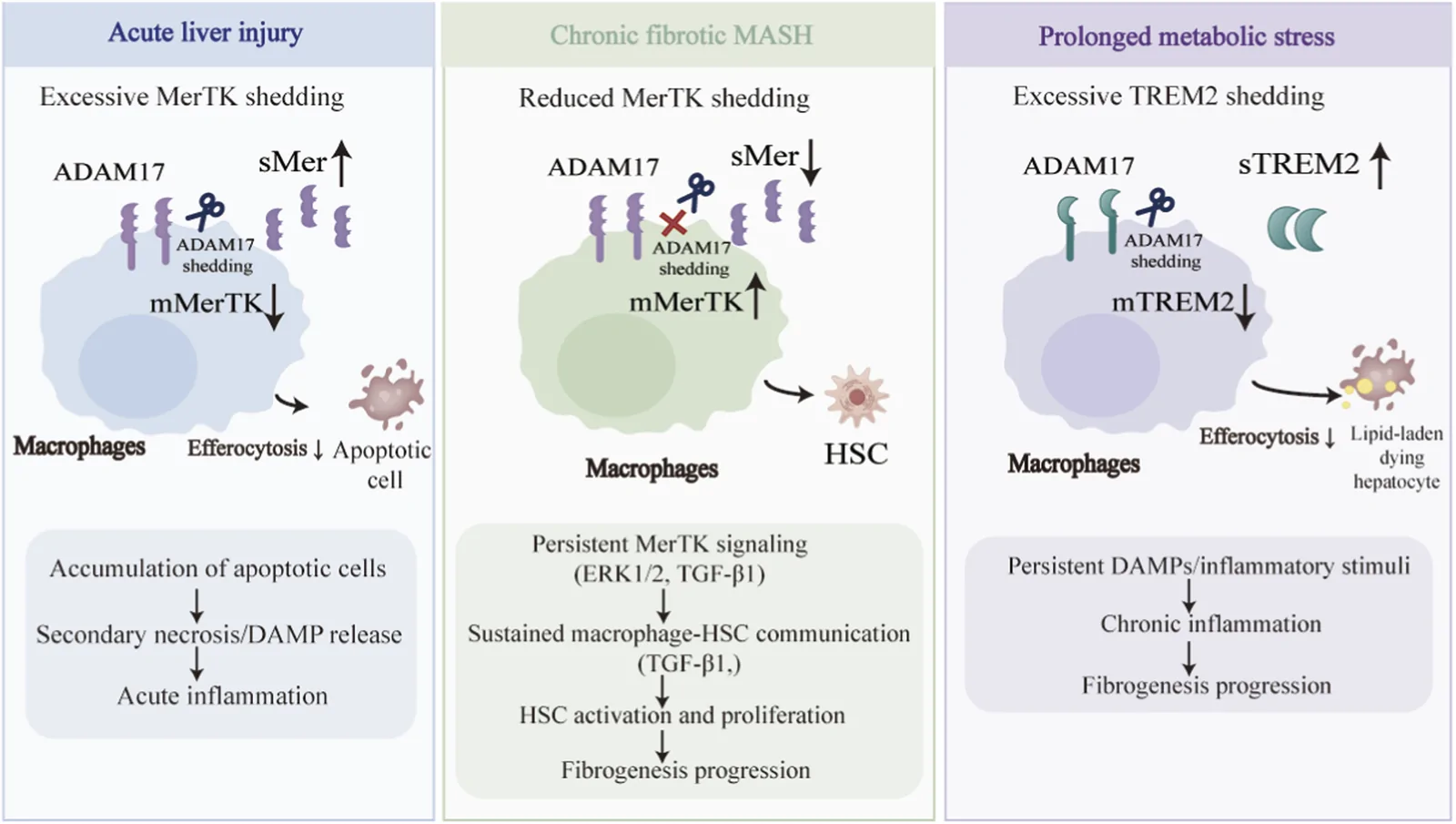
Differential ADAM17-mediated remodeling of macrophage MerTK and TREM2 during liver injury and fibrosis. Dysregulated ADAM17 activity may generate contrasting macrophage receptor states: excessive MerTK shedding during acute injury can impair efferocytosis, whereas reduced MerTK shedding in chronic MASH may sustain profibrotic signaling. Excessive TREM2 shedding reduces surface TREM2 and may compromise the clearance of lipid-laden dying hepatocytes.

The relationship between this receptor-state model and fibrosis regression is also not yet fully defined. During MASH regression, TREM2-positive lipid-associated macrophages acquire enhanced phagocytic, lipid-processing, and collagen-remodeling programs and are associated with HSC inactivation and fibrosis resolution. These observations suggest that preservation or restoration of TREM2-dependent macrophage functions may become particularly important after removal of the injurious stimulus. However, whether changes in ADAM17-mediated TREM2 shedding directly regulate these regression-associated macrophage states remains to be established.

From the perspective of inflammatory resolution, the two receptor-state abnormalities may nevertheless contribute to complementary barriers to recovery. Loss of TREM2-dependent clearance can sustain the accumulation of dying hepatocytes, DAMPs, and inflammatory stimuli, whereas persistent MerTK-associated profibrotic signaling can maintain macrophage–HSC communication. Together, these processes may hinder the transition toward reparative macrophage states and HSC inactivation. Determining whether ADAM17-mediated MerTK and TREM2 remodeling participates directly in this transition will require cell-specific, longitudinal, and spatial analyses during disease progression and regression.

## 5 ADAM17-regulated multicellular communication

At the multicellular level, the proteolytic rheostat operates by redistributing ligands and receptors between membrane-bound and soluble pools, thereby changing the direction, strength, and spatial reach of communication among hepatocytes, macrophages, HSCs, and other hepatic cells.

### 5.1 Hepatocyte–macrophage communication

During liver injury, hepatocytes and macrophages engage in bidirectional crosstalk. Injured or dying hepatocytes release DAMPs, which activate Kupffer cells and monocyte-derived macrophages. In turn, activated macrophages can either amplify hepatocyte injury or facilitate inflammatory resolution through the release of inflammatory mediators and the regulation of efferocytosis. By regulating the shedding of multiple membrane-associated mediators, ADAM17 may influence the balance between inflammatory injury, resolution, and tissue repair.

TNF-α is a representative mediator of macrophage-to-hepatocyte signaling during liver injury. Cleavage of transmembrane TNF-α expands the spatial range of TNF signaling and may amplify hepatocyte injury, whereas shedding of TNFR1/2 can limit cellular responsiveness to TNF-α (Cao et al., 2022). ADAM17-dependent release of EGFR ligands may instead activate prosurvival and regenerative signaling in hepatocytes, partially counterbalancing death receptor-mediated injury (Murthy et al., 2010).

Efferocytosis constitutes the reciprocal hepatocyte-to-macrophage arm of this communication network. Excessive shedding of MerTK or TREM2 reduces the clearance of dying hepatocytes and lipid-associated debris, allowing apoptotic material and DAMPs to persist and reinforce macrophage activation. Thus, ADAM17 regulates hepatocyte–macrophage communication not only by controlling cytokine and growth factor release but also by determining how efficiently dying hepatocytes and lipid-associated debris are cleared, thereby limiting the persistence of hepatocyte-derived DAMPs.

### 5.2 Macrophage–HSC communication

ADAM17-mediated receptor shedding also influences communication between liver macrophages and HSCs. Under endoplasmic reticulum stress, Kupffer cell-derived TNF-α can trigger apoptosis of activated HSCs through TNFR1/caspase-8 signaling, thereby limiting the accumulation of activated HSCs (Gao et al., 2020; Wang et al., 2020). Because ADAM17 regulates both TNF-α release and TNFR shedding, it could potentially modify this macrophage–HSC signaling axis. However, direct evidence that ADAM17 controls TNF-dependent HSC apoptosis *in vivo* remains limited.

Conversely, macrophage receptor-state remodeling can promote profibrotic signaling toward HSCs. In MASH, reduced MerTK shedding preserves cell-surface MerTK and sustains downstream signaling, leading to increased macrophage production of TGF-β1. Macrophage-derived TGF-β1 subsequently activates SMAD-dependent fibrogenic programs in HSCs (Cai et al., 2020). AXL, another member of the TAM receptor family, is a substrate for ADAM17-mediated ectodomain shedding. In advanced cirrhosis, GAS6 released by activated HSCs was associated with reduced AXL expression on resident liver macrophages, suggesting altered resident macrophage homeostasis (Pop et al., 2023). However, direct evidence linking this change to ADAM17-dependent AXL shedding in the liver is currently lacking.

### 5.3 Cholangiocyte, endothelial, and leukocyte compartments

Beyond hepatocyte–macrophage and macrophage–HSC communication, ADAM17 may also influence cholangiocyte, endothelial, and leukocyte compartments. However, the strength of liver-specific evidence differs substantially among these cell types. Cholangiocytes communicate with infiltrating immune cells during cholestatic liver injury. ADAM17 expression is increased in hepatocytes, cholangiocytes, and liver-infiltrating immune cells in patients with primary biliary cholangitis (PBC) or primary sclerosing cholangitis (PSC), as well as in mice with bile duct ligation (BDL). Inhibition of ADAM17 activity ameliorates cholestatic liver injury, suggesting that ADAM17 may contribute to inflammatory crosstalk between cholangiocytes and immune cells (Almishri et al., 2021).

Evidence for ADAM17-mediated receptor shedding in hepatic endothelial cells remains limited, and much of the mechanistic information currently derives from non-hepatic endothelial systems. In non-hepatic human endothelial models, TNF-α or CD40L stimulation enhances ADAM17-dependent release of soluble CD40, while ADAM17 also mediates ICAM-1 ectodomain shedding (Tsakadze et al., 2006; Klersy et al., 2023). VEGF-A has similarly been shown to promote ADAM17-dependent VEGFR2 shedding and shorten VEGFR2-mediated ERK signaling in endothelial cells (Swendeman et al., 2008). These findings provide a mechanistic basis for the hypothesis that ADAM17-dependent shedding could influence leukocyte–endothelial adhesion, transendothelial migration, and vascular signaling in the injured liver. However, direct evidence that these mechanisms operate in liver sinusoidal endothelial cells or contribute functionally to liver disease is currently lacking (Figure 4).

**FIGURE 4:**
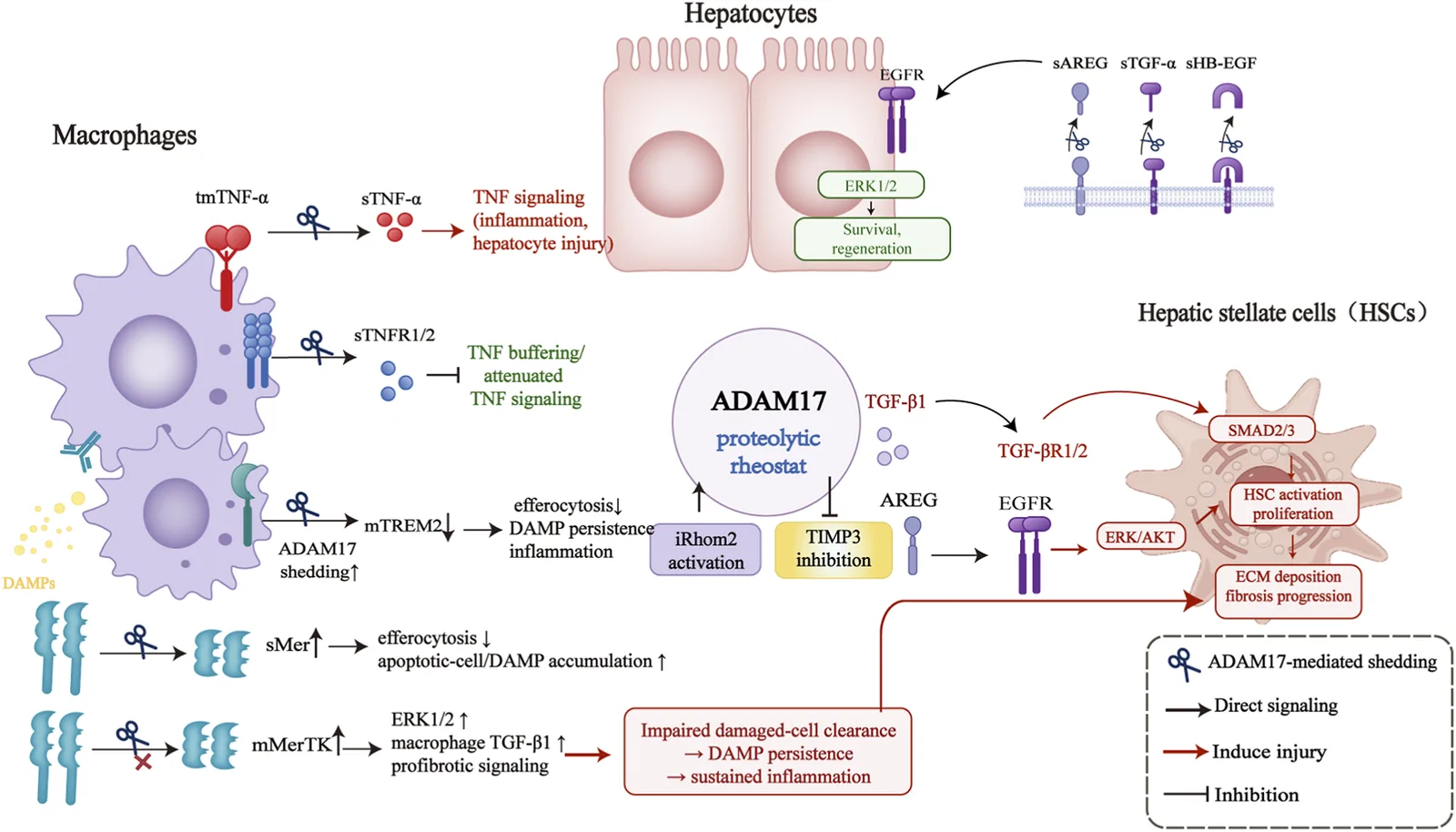
ADAM17-mediated proteolytic remodeling of macrophage–hepatocyte–HSC communication during liver injury and fibrosis. ADAM17 reshapes intercellular communication by regulating the shedding of TNF-α/TNFRs, MerTK, TREM2, and EGFR ligands. These substrate-dependent effects influence hepatocyte injury and regeneration, macrophage efferocytosis and inflammatory resolution, and macrophage–HSC profibrotic signaling. The MerTK- and TREM2-related receptor states shown here are derived from distinct experimental settings and are not established to coexist in the same macrophage population or disease stage.

## 6 ADAM17-related soluble proteins as candidate biomarkers of liver injury and fibrosis

ADAM17-mediated ectodomain shedding generates a variety of soluble proteins, some of which can be detected in the circulation. These circulating products have been associated with hepatic inflammation, fibrosis, and tissue injury and therefore represent candidate biomarkers for the noninvasive assessment of liver disease severity and progression. From a rheostat perspective, measurement of the soluble product alone captures only one side of the membrane–soluble balance. Without information on membrane-bound substrate abundance, cellular source, and competing production or clearance mechanisms, circulating soluble proteins cannot define the position of the underlying hepatic shedding rheostat.

sTNFR1/2 and sIL-6R have been evaluated as candidate circulating indicators of inflammatory activity and disease severity in chronic liver disease. In chronic hepatitis C, serum sTNFR levels correlated with histological inflammatory activity and fibrosis (Zylberberg et al., 1999). In MASH, soluble TNF receptor levels have been investigated as candidate markers of TNF-related inflammatory activity, with potential utility for fibrosis stratification (Tokushige et al., 2005; Abiru et al., 2006).

Serum sIL-6R levels are elevated in MASH, chronic HCV infection, cirrhosis, and hepatocellular carcinoma and have been associated with fibrosis severity and hepatic dysfunction. However, circulating sIL-6R is not specific to ADAM17 activity (Costantini et al., 2013). In addition to stimulus-induced ADAM17-mediated shedding, IL-6R can undergo constitutive shedding by ADAM10, and soluble IL-6R can also arise through non-proteolytic mechanisms such as alternative splicing. Interpretation of circulating sIL-6R requires particular caution because its generation is not restricted to ADAM17. ADAM17 contributes importantly to stimulated IL-6R shedding, whereas ADAM10 can mediate constitutive release, and alternative splicing can also generate soluble IL-6R isoforms. Thus, elevated circulating sIL-6R in chronic liver disease may reflect altered IL-6R biology and systemic inflammatory activity rather than hepatic ADAM17 activity alone.

Soluble TAM receptor products have been investigated as circulating biomarkers of liver disease severity and receptor shedding. In hepatitis B virus-related acute-on-chronic liver failure (HBV-ACLF), plasma MerTK levels were elevated and associated with an increased risk of short-term mortality, suggesting potential diagnostic and prognostic value (Lu et al., 2024). In MASH, serum soluble AXL (sAXL) levels increase at early disease stages. These findings support further evaluation of sAXL as an early biomarker of MASH (Tutusaus et al., 2020).

Circulating sTREM2, which can arise from proteolytic shedding of membrane TREM2 and potentially from soluble splice isoforms, has emerged as a candidate noninvasive biomarker of MASH. Circulating sTREM2 is associated with MASH, liver fibrosis, and adverse outcomes after liver surgery or transplantation, and also reflects histological improvement during treatment. Although one study reported that sTREM2 promoted CD36-dependent lipid accumulation in hepatocytes, subsequent *in vivo* evidence did not support a direct causal role for sTREM2 in fibrosing steatohepatitis (Hendrikx et al., 2022; Kothari et al., 2023). Thus, sTREM2 is currently better regarded as a candidate biomarker of liver disease activity and severity than as an established pathogenic mediator (Indira Chandran et al., 2023; Peng et al., 2025; Santol et al., 2025; Wernberg et al., 2025; Dempsey et al., 2026). However, circulating sTREM2 should not be considered a direct measure of hepatic ADAM17 activity. TREM2 can be cleaved by both ADAM10 and ADAM17, soluble TREM2 isoforms can also arise through alternative splicing in human systems, and circulating sTREM2 may originate from myeloid cells outside the liver. The relative contribution of these sources in liver disease remains unresolved. Similar considerations apply to sTNFR1/2, sMer, and sAXL. Their circulating concentrations may be influenced by substrate expression and turnover, proteolytic release from hepatic or extrahepatic cells, and systemic clearance, and therefore should not be interpreted as direct measures of hepatic ADAM17 activity.

HB-EGF has also been investigated as a candidate circulating biomarker. Plasma HB-EGF levels increased after major partial hepatectomy, and higher peak concentrations were associated with greater increases in remnant liver volume, suggesting a potential relationship with the postoperative regenerative response (Yamada et al., 1998). In patients with chronic hepatitis C, lower circulating HB-EGF concentrations were associated with greater liver disease severity (Bocci et al., 2022).

In summary, several ADAM17-related shedding products, including sTNFR1/2, sIL-6R, sMer, sAXL, sTREM2 and HB-EGF, have been associated with fibrosis severity, inflammatory activity, or clinical outcomes across multiple liver diseases. Some analytes, particularly sTREM2 and sAXL, may change at relatively early stages of disease (Table 2). Collectively, these molecules are therefore better regarded as candidate readouts of liver injury, inflammatory burden, or receptor-state remodeling than as direct surrogates for hepatic ADAM17 activity. Establishing their value as biomarkers of hepatic ADAM17 activity will require paired blood–tissue studies integrating circulating soluble products with hepatic ADAM17 activity, membrane-bound substrate abundance, and cell-of-origin analysis.

**TABLE 2:** Candidate circulating biomarkers related to ADAM17-regulated ectodomain shedding in liver disease.

Soluble analyte	Liver disease or clinical setting	Reported clinical association	Major limitation	References
sTNFR1/2	Chronic HCV infection and MASH	Associated with histological inflammatory activity, fibrosis and indices of disease severity	Receptor expression, tissue source and clearance	Zylberberg et al., 1999; Tokushige et al., 2005; Abiru et al., 2006
sIL-6R	MASH, chronic HCV infection, cirrhosis and HCC	Elevated circulating levels; associated with fibrosis stage and Child–Pugh score	Multiple cellular sources and production mechanisms	Shipovskaya et al., 2025; Migita et al., 2006; Costantini et al., 2013
Plasma MerTK/sMer	HBV-related acute-on-chronic liver failure	Elevated levels associated with diagnosis and short-term mortality	Limited clinical data; paired tissue validation lacking	Lu et al., 2024
sAXL	MASH	Elevated at relatively early disease stages	Not liver-specific; affected by receptor expression, turnover, and shedding	Tutusaus et al., 2020
sTREM2	MASLD/MASH, liver surgery, liver transplantation	Associated with MASH/fibrosis, perioperative outcomes, and treatment response	Not liver-specific; direct pathogenic role remains unproven	Hendrikx et al., 2022; Kothari et al., 2023; Indira Chandran et al., 2023; Peng et al., 2025; Santol et al., 2025; Wernberg et al., 2025; Dempsey et al., 2026
HB-EGF	Major hepatectomy; chronic HCV infection	Associated with postoperative liver-volume recovery and HCV disease severity	Circulating levels do not distinguish synthesis from ADAM17-dependent shedding	Yamada et al., 1998; Bocci et al., 2022

## 7 Therapeutic targeting of the ADAM17 shedding axis

### 7.1 Direct inhibition of ADAM17

Direct inhibition of ADAM17 catalytic activity represents the most straightforward pharmacological approach to limiting pathological ectodomain shedding. Early strategies largely relied on small-molecule metalloprotease inhibitors that targeted the catalytic site. In a bile duct ligation model of cholestatic liver injury, pharmacological inhibition of ADAM17 with DPC333 significantly reduced biochemical and histological liver injury and attenuated disease-associated behavioral changes (Almishri et al., 2021). Notably, ADAM17 expression was also increased in hepatocytes, cholangiocytes, and liver-infiltrating immune cells in liver biopsies from patients with primary biliary cholangitis and primary sclerosing cholangitis, providing clinical context for this experimental proof of concept. However, these findings are confined largely to cholestatic injury, and whether direct catalytic inhibition is beneficial in other forms or stages of chronic liver disease remains uncertain.

A major limitation of conventional small-molecule inhibitors is selectivity. Because the catalytic clefts of ADAM and matrix metalloproteinase family members are structurally related, early compounds frequently inhibited multiple metalloproteases (Peng et al., 2016). Apratastat, for example, is a dual TACE/MMP inhibitor that effectively suppressed TNF-α release in pharmacodynamic studies but failed to demonstrate clinical efficacy in rheumatoid arthritis despite adequate target engagement (Shu et al., 2011). This experience illustrates that biochemical inhibition of ADAM17 does not necessarily translate into therapeutic benefit and that broad metalloprotease inhibition may complicate both efficacy and safety.

More selective biological approaches have therefore been developed. The monoclonal antibody MEDI3622 recognizes a unique surface region of the ADAM17 metalloprotease domain rather than the conserved catalytic cleft, allowing highly selective inhibition of ADAM17-dependent shedding (Peng et al., 2016). In preclinical tumor models, MEDI3622 suppressed ADAM17-dependent signaling and inhibited tumor growth (Rios-Doria et al., 2015), while other inhibitory antibodies, including D1 (A12) and D8P1C1, similarly reduced ADAM17-mediated shedding of TNF-related or EGFR-family substrates (Richards et al., 2012; Huang et al., 2014; Saha et al., 2022). However, these studies were conducted predominantly in non-hepatic systems and should therefore be regarded as evidence for the pharmacological tractability and selectivity of ADAM17, rather than direct evidence of therapeutic efficacy in liver disease. Whether antibody-based ADAM17 inhibition can achieve sufficient hepatic exposure and preserve beneficial shedding responses during chronic liver injury remains to be determined.

### 7.2 Targeting the TIMP3–ADAM17 regulatory axis

An alternative to direct catalytic inhibition is to modulate the endogenous TIMP3–ADAM17 regulatory axis. TIMP3 is an extracellular inhibitor of ADAM17 and can restrain the shedding of inflammatory cytokines, receptors, and growth-factor precursors. Liver-related studies support the therapeutic relevance of restoring TIMP3 activity. Macrophage-specific TIMP3 overexpression protected mice from diet-induced metabolic inflammation, improved insulin sensitivity, and reduced inflammatory and oxidative stress responses in the liver, suggesting that enhancement of TIMP3 may be beneficial in metabolic liver disease (Menghini et al., 2012). More recent evidence also links increased TIMP3 expression with protection against hepatic ischemia–reperfusion injury, although direct involvement of ADAM17 was not established in that study. Importantly, TIMP3 is not an ADAM17-specific inhibitor and can regulate other extracellular metalloproteases. Therefore, the effects of TIMP3 restoration cannot be attributed exclusively to reduced ADAM17 activity. Moreover, excessive suppression of ADAM17 could interfere with protective shedding events, including TNFR release and EGFR-ligand-mediated tissue repair. Thus, selective or temporally controlled modulation of the TIMP3–ADAM17 balance may be preferable to sustained systemic enhancement of TIMP3.

### 7.3 Targeting iRhom2-dependent ADAM17 regulation

Targeting iRhom2 provides an alternative strategy for modulating ADAM17 without directly blocking its catalytic site. As a regulatory partner of ADAM17, iRhom2 controls its maturation, stimulus-dependent activation, and substrate selectivity, raising the possibility of more context- or substrate-selective modulation of ADAM17 shedding. Liver-specific studies, however, reveal markedly context-dependent effects. iRhom2 deficiency attenuated inflammation, oxidative stress, and fibrosis in experimental alcohol-associated liver injury, supporting its potential as a therapeutic target in inflammatory liver disease (Liu et al., 2022). Similar protective effects were observed in PM2.5-induced hepatic steatosis and dyslipidemia (Ge et al., 2017).

In contrast, iRhom2 deficiency aggravated bile duct ligation-induced fibrosis by reducing ADAM17-dependent TNFR1/2 shedding (Sundaram et al., 2019), indicating that iRhom2 can also support protective TNF-receptor regulation. These opposing findings argue against indiscriminate iRhom2 inhibition. Although genetic studies support the therapeutic relevance of the iRhom2–ADAM17 axis, pharmacological strategies capable of selectively modulating hepatic iRhom2–ADAM17 signaling remain at an early stage. RNA-based approaches, including antisense oligonucleotides or siRNA, and strategies that interfere with stimulus-dependent iRhom2–ADAM17 interactions are conceptually attractive, but their liver-specific efficacy, delivery, substrate selectivity, and safety remain to be established. Future interventions may therefore need to target specific iRhom2–ADAM17 activation states rather than suppressing iRhom2 globally.

### 7.4 Cell-, substrate-, and stage-selective targeting

The pleiotropic functions of ADAM17 suggest that future therapeutic strategies may need to move beyond global inhibition toward cell-, substrate-, and disease-stage-selective modulation. Cell-selective approaches could preferentially suppress pathogenic ADAM17 activity in inflammatory macrophages or other disease-driving compartments while preserving its physiological functions elsewhere. Targeting components of the ADAM17 sheddase complex may provide one route toward such compartmental control; for example, modulation of iTAP/FRMD8 has been proposed to restrict ADAM17 activity in selected cellular compartments without reproducing the broader consequences of global ADAM17 deficiency (Badenes et al., 2023). Substrate-selective targeting represents another emerging strategy. A recently developed MerTK-binding nanobody selectively prevented MerTK ectodomain cleavage without interfering with ligand binding or kinase activity, providing proof of concept that pathological shedding of an individual ADAM17 substrate can be selectively blocked, although this approach has not yet been evaluated in liver disease (Duprez et al., 2026).

Finally, the timing of intervention may be equally important. ADAM17-mediated MerTK cleavage can impair macrophage efferocytosis during acute liver ischemia–reperfusion injury, and pharmacological ADAM17 inhibition restored efferocytosis and reduced liver injury in this setting (Guan et al., 2026). Conversely, ADAM17-dependent release of EGFR ligands can activate regenerative MAPK and PI3K/Akt signaling in hepatocytes, illustrating the potential benefit of preserving ADAM17 activity during tissue repair (Voigt et al., 2026). Thus, therapeutic intervention may ultimately require matching the targeted substrate and cell type to the dominant pathological process and stage of liver injury.​These observations suggest that the therapeutic window for ADAM17 modulation is likely to be disease-stage dependent and should be defined experimentally before stage-specific interventions can be translated clinically.

## 8 Discussion

The transition from liver injury to fibrosis is not dictated by a single proinflammatory or profibrotic signal; rather, it reflects the dynamic interplay among persistent inflammation, inflammatory resolution, tissue repair, and multicellular signaling. ADAM17 should be viewed not only as the TNF-α-converting enzyme but also as a proteolytic regulator within the hepatic injury microenvironment. By shedding membrane-bound substrates, including TNF-α/TNFRs, IL-6R, EGFR ligands, MerTK, and TREM2, ADAM17 alters the balance between membrane-bound and soluble molecular forms, thereby shaping inflammatory signaling, reparative responses, and fibrotic remodeling.

A temporal perspective helps reconcile several apparently opposing effects of ADAM17 described in this review. During acute injury, ADAM17-mediated shedding participates in rapid inflammatory amplification but can simultaneously activate counter-regulatory and hepatoprotective pathways. During the subsequent resolution and regenerative phase, efferocytic receptors and EGFR ligands contribute to the clearance of damaged cells and restoration of tissue integrity. If injury persists, however, prolonged or imbalanced shedding can favor chronic inflammation, loss of pro-resolving receptor states, and macrophage–HSC profibrotic communication. Established fibrosis therefore reflects not simply increased ADAM17 activity, but a persistent maladaptive pattern of substrate processing. During fibrosis regression, macrophage programs associated with efferocytosis, lipid handling, and matrix remodeling may again become dominant, although the contribution of ADAM17-dependent shedding to this phase remains less well defined.

ADAM17-mediated remodeling of macrophage receptor states may be an important determinant of liver injury outcomes. Reduced MerTK shedding preserves cell-surface MerTK and may sustain the MerTK/ERK/TGF-β1 signaling axis, thereby promoting profibrotic crosstalk between macrophages and HSCs. Conversely, excessive TREM2 shedding may reduce cell-surface TREM2 availability, compromise the clearance of apoptotic hepatocytes and lipid-associated debris, and sustain DAMP-associated inflammatory signaling. Reduced MerTK shedding and excessive TREM2 shedding represent two distinct, independently supported patterns of macrophage receptor-state dysregulation. Their comparison suggests that both insufficient and excessive ectodomain shedding can be maladaptive, depending on the substrate. However, these observations should not be interpreted as evidence for a unified MerTK–TREM2 pathway. Whether the two receptor states coexist within the same macrophage population, arise sequentially during disease progression, or occur in distinct spatial and temporal niches remains unknown.

The proteolytic rheostat framework extends the general concept of context dependence in four ways. First, it focuses on the relative distribution of each substrate between membrane-bound and soluble states rather than on total ADAM17 abundance. Second, it recognizes that simultaneous cleavage of different substrates can produce opposing biological outputs. Third, it incorporates spatial and temporal dimensions of signaling, because shedding can alter both the range and duration of intercellular communication. Fourth, it predicts that pathology may arise from either excessive or insufficient cleavage, depending on the substrate and disease stage.

Future studies should integrate cell type-specific genetic models with single-cell and spatial omics approaches to define the substrate selectivity of ADAM17 across hepatic cellular compartments and to resolve its temporal and spatial dynamics during disease progression. Soluble shedding products, including sTNFR, sIL-6R, sMer, sTREM2, and sAXL, may serve as candidate circulating readouts of liver injury and fibrosis; however, they should not be interpreted as direct surrogates for hepatic ADAM17 activity. Rather than broadly inhibiting ADAM17, future therapeutic strategies may benefit from cell type, disease stage, or substrate selective interventions that preserve its inflammation-limiting and tissue-repair functions while attenuating its profibrotic effects.
